# β-cell-targeted RNA activation of vascular endothelial growth factor-A improves islet transplantation

**DOI:** 10.1038/s41392-026-02934-8

**Published:** 2026-08-04

**Authors:** Dimitri Van Simaeys, Per-Olof Berggren

**Affiliations:** 1 The Rolf Luft Research Center for Diabetes and Endocrinology, Karolinska Institutet, Stockholm, Sweden; 2 Diabetes Research Institute, University of Miami Miller School of Medicine, Miami, FL USA; 3 West China Hospital, Sichuan University, Chengdu, China; 4 Tecnológico de Monterrey, School of Medicine and Health Sciences, Monterrey, Mexico

**Keywords:** Endocrine system and metabolic diseases, Molecular medicine, Translational research, Preclinical research

## Abstract

Pancreatic islet transplantation can restore insulin production in patients with severe diabetes, but donor material is scarce, and early engraftment is constrained by inflammatory and mechanical stress and a prolonged avascular phase, during which oxygen and nutrient delivery are limited by diffusion. Revascularization relies primarily on stress-induced vascular endothelial growth factor A (VEGF-A), prolonging metabolic compromise, increasing autophagic burden, and rendering grafts vulnerable to secondary stress. Accelerating vascular integration during this time window is therefore critical for graft health. We show that β-cell-targeted aptamer-VEGF-A small activating RNA (saRNA) chimeras selectively induce robust VEGF-A expression in mouse and human islets independently of hypoxia- or nutrient-stress pathways, without activating autophagy or stress-responsive genes. In vivo, chimera-primed islets transplanted into anterior chamber and kidney capsule models exhibited accelerated vascular migration, earlier perfusion, and faster resolution of LC3-dependent autophagic stress without altering endpoint vascular density. Functionally, marginal-mass mouse and human grafts restored glucose control more effectively, preserved intra-islet architecture, and delayed hyperglycemia following STZ-induced β-cell loss. The RNA chimera’s modular architecture of RNA chimeras allows transient, tissue-adaptable transcriptional activation across species and cell sources. These findings establish that brief, ex vivo RNA-mediated priming preconditions islets to withstand early engraftment stress, enhancing vascular integration and functional outcomes. This scalable, stress-independent strategy may lower the minimum effective transplant mass and expand access to cellular therapies for diabetes.

## Introduction

Pancreatic islet transplantation has matured from an experimental procedure into an established cell therapy that can restore endogenous insulin secretion and protect against severe hypoglycemia in selected patients with type 1 diabetes.^1,2^ Following the Edmonton protocol, incremental gains in isolation, immunosuppression, and recipient selection have steadily increased the proportion of recipients achieving insulin independence. More recently, the field has begun moving toward renewable, stem-cell-derived β-cell sources, broadening islet therapy beyond its historical cadaveric supply.^3–6^ Regardless of cell source, however, the engraftment period remains the central determinant of long-term function. Islets are stripped of their native vasculature during isolation from the donor pancreas, and they remain avascular after implantation, entering a prolonged phase in which oxygen and nutrient delivery depend entirely on passive diffusion.^7,8^ During this window, they are exposed to instant blood-mediated inflammatory injury, release of damage-associated molecular patterns, and progressive hypoxic and nutrient stress, which together drive substantial early β-cell loss.^9,10^ Functional recovery hinges on revascularization, a process largely governed by vascular endothelial growth factor A (VEGF-A) and essential for rebuilding the dense intra-islet capillary network.^7,8^ Numerous strategies, including alternative implantation sites, anti-inflammatory conditioning, and vascular-targeted interventions, have been explored to shorten this vulnerable interval, with only partial success.^11–16^

Type 1 diabetes affects approximately 9 million people worldwide, and its incidence continues to rise.^17^ For the subset with hypoglycemia unawareness or brittle disease refractory to optimized medical management, islet transplantation remains the only intervention capable of restoring physiological glucose sensing.^18,19^ Its clinical reach is nonetheless constrained by the scarcity of donor tissue and by the inefficiency of engraftment itself.^20^ Because a large fraction of the transplanted mass is lost in the first days after implantation, clinical protocols compensate by infusing very high numbers of islet equivalents, frequently pooling preparations from two or more donors per recipient.^20^ Any intervention that improves early survival and accelerates revascularization would therefore lower the minimum effective transplant mass, reduce the donor burden, and broaden access to a therapy currently rationed by supply.^21^ The engraftment bottleneck is thus not merely a biological curiosity but the principal barrier to scaling islet replacement.

The difficulty is that VEGF-A induction is, by default, a stress response. Under physiological conditions, its transcription is coupled to hypoxia- and nutrient-sensing pathways, so the angiogenic signal is generated only once the graft is already metabolically compromised.^22–25^ This coupling creates a self-defeating sequence: the very deprivation that should trigger revascularization simultaneously activates autophagic and integrated stress programs that erode β-cell viability, so vascular rescue arrives only after substantial, often irreversible, attrition.^22–24,26–28^ Decoupling VEGF-A expression from this cascade, by inducing it pre-emptively before deprivation sets in, could in principle break the sequence and preserve functional mass. Efforts to supply VEGF-A exogenously have thus far fallen short of this goal. Viral and nonviral gene transfer,^29–31^ VEGF-secreting engineered cells,^21,32^ and polymer- or hydrogel-based delivery systems^33,34^ each provide proof-of-concept benefit but suffer from limited spatial specificity, unstable or unregulated expression, and a risk of aberrant angiogenesis from sustained overexpression.^35^ Direct mRNA delivery offers transient, integration-free protein production^36–38^ and has been applied to islet revascularization,^37^ yet efficient, β-cell-selective delivery remains constrained by stability, cellular uptake, and intracellular trafficking.^39–43^ What has been missing is a way to raise VEGF-A locally, transiently, and selectively within the islet, on demand and ahead of stress, without permanent genetic modification.

Here, we address this gap with a modular aptamer–saRNA chimera that activates the endogenous VEGF-A locus directly within β-cells. Small activating RNAs (saRNAs) are short duplexes that, through an Argonaute-guided mechanism, recognize a target gene’s promoter in a sequence-specific manner and stimulate transcription of that single endogenous gene rather than silencing it, affording transient, gene-specific upregulation without genomic integration.^44–46^ We couple a human- or mouse-specific VEGF-A saRNA^47^ to a β-cell-targeting aptamer delivery module,^48–51^ rendering expression both cell-selective and uncoupled from hypoxia- and nutrient-stress signaling.^25^ Applying this strategy to islet transplantation for the first time, we show that a brief, ex vivo priming step drives robust, stress-independent VEGF-A expression in both mouse and human islets, accelerating graft revascularization and perfusion, compressing the early wave of autophagic stress, and preserving functional β-cell mass. In marginal-mass models in the anterior chamber of the eye and under the kidney capsule, primed grafts restored glucose control more effectively and delayed hyperglycemia, establishing a scalable route to improved engraftment across species and cell sources.

## Results

### β-cell-binding aptamer-VEGF-A saRNA chimera induces robust VEGF-A expression in islets

**Fig. 1:**
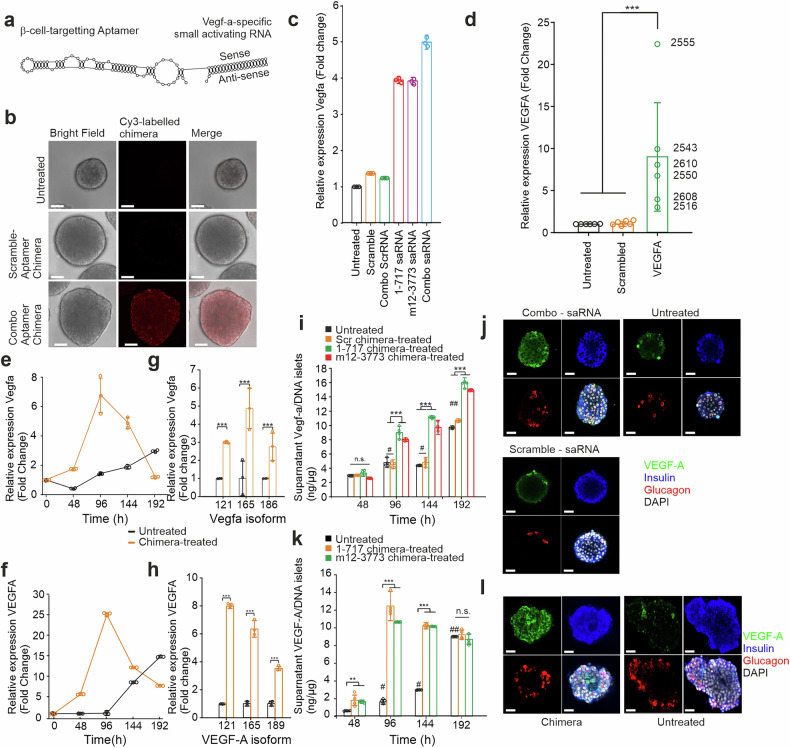
β-cell-targeting aptamer–saRNA chimera induces VEGF-A expression in mouse and human islets. **a** Schematic of the aptamer–saRNA chimera showing the β-cell-targeting aptamer and species-specific VEGF-A saRNA. **b** Confocal images showing uptake of Cy3-labeled chimera in whole mouse islets after 1 h incubation; scramble aptamer-saRNA chimera control shown for comparison. **c** Relative VEGF-A mRNA expression in mouse islets treated with 1-717-, m12-3773- and both (combo)-VEGF-A and scramble saRNA chimera mix, scramble aptamer VEGF-A VEGF-A saRNA control, or untreated, after 96 h measured by qPCR. **d** VEGF-A induction across six human donors, demonstrating robust upregulation compared with untreated islets. Representative time course of VEGF-A mRNA induction in mouse (**e**) and human (**f**) islets, comparing untreated and chimera-treated groups. Isoform-specific transcriptional analysis in mouse (**g**) and human (**h**) islets after 96 h of chimera treatment, showing upregulation across multiple VEGF-A isoforms. ELISA time course of VEGF-A secretion in mouse (**i**) and human (**k**) islets treated with individual aptamer–saRNA chimeras compared with mock treatment; values normalized to total islet DNA (*n* = 4). Confocal imaging of VEGF-A protein induction 96 h after combo chimera treatment in mouse (**j**) and human (**l**) islets, highlighting β-cell-specific upregulation; untreated (**j**, **l**) and scramble (**j**) controls are shown for comparison. Mean ± SD; *: *P* < 0.05, **: *P* < 0.01, ***: *P* < 0.001 versus untreated or scramble controls. Scale bars: 25 µm (**j**–**l**), 40 µm (**b**)

To determine whether a β-cell-targeting aptamer-VEGF-A saRNA chimera can upregulate VEGF-A, we conjugated VEGF-A-specific small activating RNAs (saRNAs) to two validated β-cell-binding RNA aptamers (1-717 and m12-3773) recognizing the cell-surface proteins TMED6 and CLU in mouse and human islets, respectively (Fig. 1a).^51^ Species-specific saRNAs were used to accommodate differences in VEGF-A promoter sequences.^44,52^

Confocal imaging of Cy3-labeled chimeras confirmed efficient uptake in mouse islets via aptamer-mediated internalization, while scrambled controls showed minimal uptake (Fig. 1b). Quantitative PCR demonstrated robust VEGF-A transcription in mouse islets treated with either aptamer chimera, with further enhancement observed when both aptamers were combined (Fig. 1c). Specificity controls, including scrambled aptamer or scrambled saRNA combinations, exhibited negligible induction. In human islets from six donors, chimera treatment consistently increased VEGF-A transcripts compared with untreated controls (Fig. 1d).

Time-course experiments revealed sustained VEGF-A mRNA induction in both species, peaking at 96 h posttreatment and gradually returning toward baseline by 192 h (Fig. 1e, f). Isoform-specific analysis confirmed the upregulation of multiple canonical VEGF-A isoforms, indicating physiologically relevant transcriptional activation (Fig. 1g, h). ELISA confirmed early VEGF-A secretion in chimera-treated islets, with untreated islets reaching similar elevated levels only after extended culture, likely reflecting stress-mediated VEGF-A induction^24,53^ (Fig. 1i, k). The absence of early secretory induction in scramble-chimera controls confirms that VEGF-A upregulation is sequence-specific and not attributable to the aptamer delivery module. Confocal immunostaining corroborated these findings, i.e., increased VEGF-A protein was detected in insulin-positive β-cells of chimera-treated mouse and human islets, while scramble-chimera-treated islets displayed staining patterns comparable to those of untreated controls (Fig. 1j, l). Chimera treatment did not impair β-cell secretory function, as glucose-stimulated insulin secretion was preserved across all stimulation conditions, tested 96 h after chimera addition (Supplementary Fig. 1).

Together, these data demonstrate that β-cell-targeting aptamer-saRNA chimeras enable selective, transient, and robust VEGF-A induction in mouse and human islets, providing a platform for enhancing vascularization in transplanted grafts.

### SaRNA_VEGF-A_ aptamer chimera enhances revascularization following syngeneic mouse islet transplantation

**Fig. 2:**
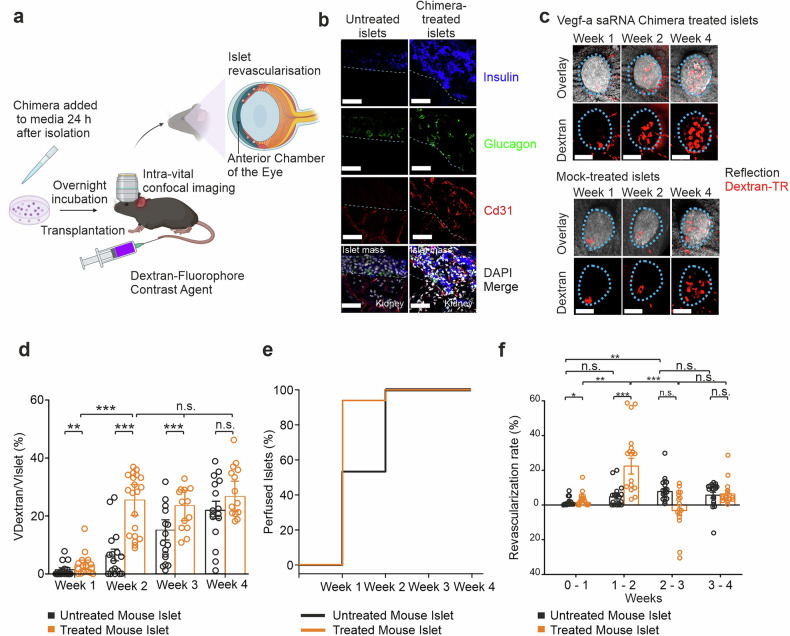
Aptamer–saRNA priming accelerates islet revascularization in vivo. **a** Schematic overview of the experimental workflow for mouse islet preparation, chimera treatment, and transplantation into the kidney capsule or ACE. Created with BioRender.com. **b** Confocal images of syngeneic mouse islet grafts transplanted under the kidney capsule one week post-transplantation, comparing mock-treated and chimera-treated islets. Blood vessels are visualized by Cd31 staining, Scale bar = 50 μm. **c** Representative top-view confocal projections of mouse islets transplanted into the ACE at weeks 1, 2, and 4 following transplantation. Chimera-treated and mock-treated grafts are shown side-by-side. Functional perfusion is visualized by dextran influx (red) and reflection imaging (gray). **d** Longitudinal tracking of individual ACE-transplanted islets over time, showing intra-islet vascular progression in chimera-treated versus mock-treated grafts. Each trajectory represents a single islet followed across multiple imaging sessions. **e** Kaplan–Meier analysis of functional reperfusion, defined by the appearance of dextran-filled intra-islet vessels, in chimera-treated and mock-treated islets over the monitoring period (*P* < 0.001, log-rank test). **f** Relative rate of revascularization derived from the longitudinally tracked islets in (**d**), calculated as the change in normalized vascular perfusion between imaging intervals. Chimera-treated islets exhibit a concentrated revascularization phase predominantly during week 2 post-transplantation, whereas mock-treated islets display a more gradual and distributed revascularization profile. Each data point represents an individual islet (5 mice per group; 3–8 islets per mouse). Data are shown as mean ± SD. Statistical comparisons were performed using two-way ANOVA or log-rank test as appropriate. *P < 0.05, **P < 0.01, ***P < 0.001

To evaluate whether VEGF-A upregulation by the β-cell-targeting aptamer–saRNA chimera accelerates functional vascular integration, isolated mouse islets were incubated with chimera or mock-treated control for 24 h prior to transplantation (Fig. 2a). Pretreatment did not induce observable morphological changes. As expected, endothelial cells were largely lost during isolation, confirming that subsequent vessel formation derives from host vasculature (Supplementary Fig. 2).

Initial benchmarking under the kidney capsule revealed that chimera-treated grafts exhibited enhanced insulin staining and more extensive CD31^+^ vascularization one week after transplantation compared with mock-treated islets (Fig. 2b), indicating that VEGF-A priming promotes early graft reperfusion by host vessel recruitment.

To enable longitudinal and high-resolution tracking of revascularization, we transplanted chimera- or mock-treated islets into the anterior chamber of the eye (ACE), a site that allows noninvasive imaging and partial immune isolation (Fig. 2a). Weekly intravital confocal imaging revealed that chimera-treated grafts showed dextran-perfused vessels as early as one week post-transplantation, whereas mock-treated islets exhibited delayed perfusion, typically appearing by week 2 (Fig. 2c–e). Vascular ingrowth in chimera-treated islets progressed rapidly from the islet base to the apex, with near-complete integration by week 4, whereas control grafts displayed more gradual vascularization.

Importantly, quantitative analysis of longitudinally tracked islets demonstrated that the chimera primarily accelerated the rate of revascularization rather than altering the final vascular endpoint (Fig. 2f). Specifically, chimera-treated islets underwent the most vascular integration during week 2, while mock-treated islets displayed a more delayed revascularization profile. These findings highlight that early VEGF-A induction by aptamer–saRNA chimeras effectively primes islets to engage host vasculature sooner, providing a mechanistic basis for improved graft reperfusion.

### VEGF-A chimera priming enhances functional recovery and preserves architecture in marginal-mass syngeneic transplantations

**Fig. 3:**
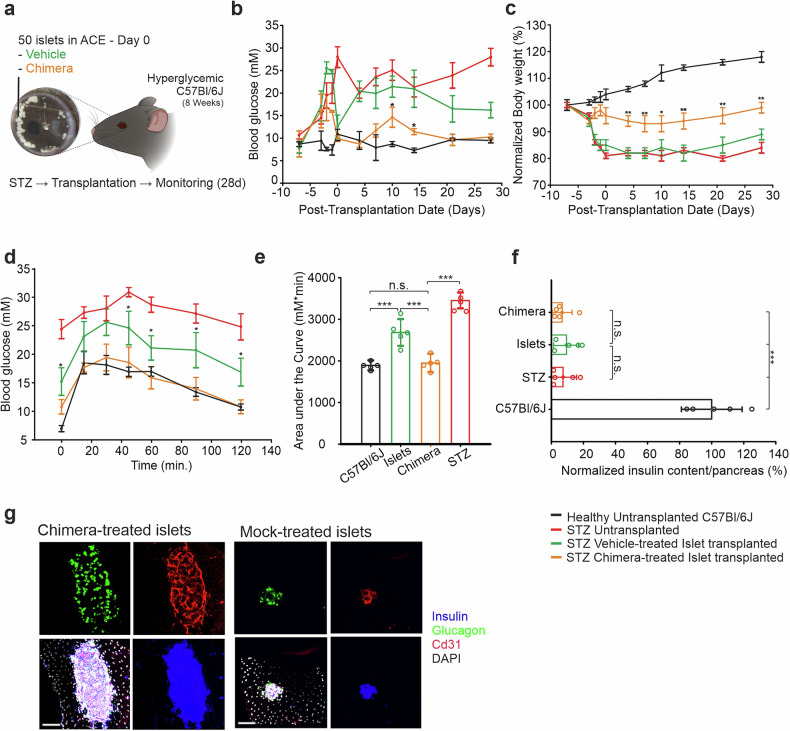
VEGF-A chimera islet priming improves islet graft function in syngeneic marginal mass transplantations in mice and preserves graft architecture. **a** Schematic illustration of marginal-mass mouse islet transplantation into the ACE and longitudinal metabolic monitoring. Created with BioRender.com. **b** Blood glucose levels over time following transplantation of chimera-treated or mock-treated mouse islets. Normoglycemia is defined as blood glucose <16 mM. Healthy and STZ-only mice are shown as reference controls. **c** Body weight monitoring of transplanted mice over the same period. **d** Intraperitoneal glucose tolerance test (IPGTT) performed 28 days post-transplantation. **e** Area under the curve (AUC) analysis of IPGTT responses shown in (**d**). **f** Pancreatic insulin content at the study endpoint, normalized to healthy controls. **g** Representative whole-mount confocal projections of explanted islet-iris grafts showing preservation of intra-islet architecture in chimera-treated grafts compared with mock-treated grafts. Insulin (blue), Glucagon (green), Cd31 (red), and DAPI (gray). Data are presented as the mean ± SEM (**b**, **c**) and as the mean ± SD in (**d**, **e**, **f**). *n* = 5 (healthy/STZ controls), *n* = 8 (chimera-treated and mock-treated islet groups) in (**b**, **c**); *n* = 5 in (**d**, **e**, **f**). Statistical comparisons were performed using two-way ANOVA or one-way ANOVA with post hoc testing as appropriate. *P < 0.05, **P < 0.01, ***P < 0.001

To determine whether chimera-mediated VEGF-A induction improves functional outcomes in a stringent transplantation setting, we transplanted a submarginal mass of 50 mouse islets into the ACE of STZ-treated hyperglycemic C57Bl/6J mice (Fig. 3a). Blood glucose and body weight were monitored for 28 days posttransplantation. Recipients of chimera-treated islets regained normoglycemia (<16 mM) by day 21, whereas mock-treated controls remained hyperglycemic (Fig. 3b). Chimera-treated mice also maintained body weights comparable to those of healthy controls, in contrast to the initial weight loss observed in mock-treated or STZ-only mice (Fig. 3c).

Metabolic assessment at the study endpoint demonstrated improved glucose handling in chimera-treated recipients. Intraperitoneal glucose tolerance testing (IPGTT) revealed that chimera-treated mice exhibited glucose excursions similar to those of healthy controls, whereas mock-treated mice displayed impaired glucose tolerance (Fig. 3d), which was confirmed by area-under-the-curve analysis (Fig. 3e). Pancreatic insulin content remained similarly low in all STZ-treated groups (10–15% of healthy controls), indicating that glycemic recovery in chimera-treated mice was attributable to the transplanted islets (Fig. 3f).

Histological analysis of explanted grafts revealed preservation of intra-islet architecture in chimera-treated islets. Confocal whole-mount imaging showed β-cell-rich cores surrounded by α-cells and a dense, reticular vascular network. In contrast, mock-treated grafts displayed reduced β-cell volume, disorganized vascular networks, and morphological signs of cellular stress (Fig. 3g).

Together, these findings demonstrate that VEGF-A chimera pre-treatment accelerates functional reperfusion and preserves graft cytoarchitecture in marginal-mass islet transplants, supporting its potential to enhance transplantation outcomes under clinically relevant limiting conditions.

### Early VEGF-A induction reduces autophagic stress and preserves islet integrity

Following transplantation, pancreatic islets experience a transient but critical avascular phase in which oxygen and nutrient delivery are limited by diffusion. This period imposes metabolic stress on the graft and has been proposed to trigger autophagy and structural remodeling until host-derived vessels are established.^24,26,27^ We hypothesized that accelerating revascularization would shorten this stress window and thereby preserve islet integrity during early engraftment. To test this hypothesis, we first modeled nutrient-limited conditions in vitro to define how isolated islets respond to sustained diffusion constraints and then examined whether chimera-mediated VEGF-A induction could mitigate stress responses during transplantation in vivo.

**Fig. 4:**
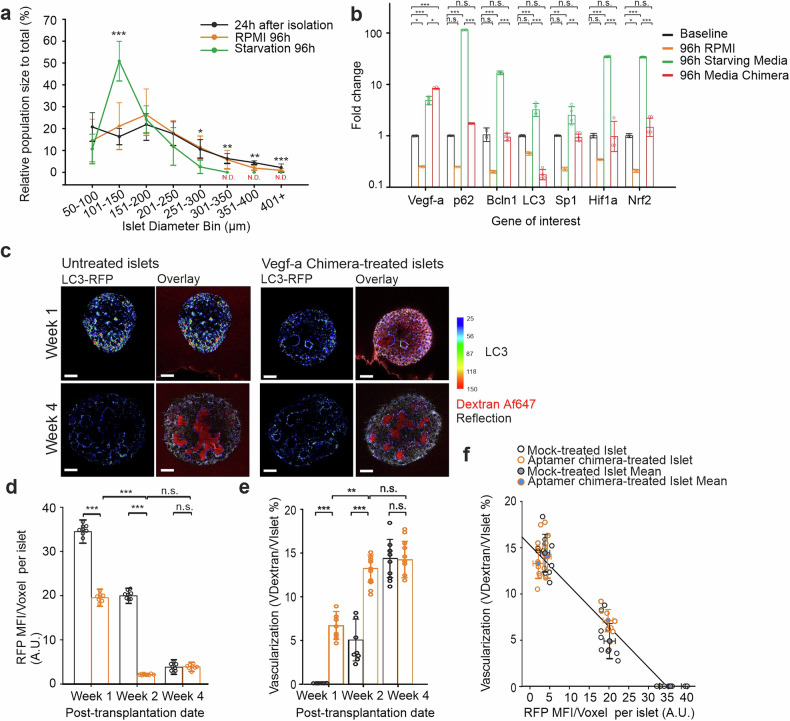
VEGF-A induction bypasses stress-associated pathways and accelerates stress resolution in transplanted islets. **a** Relative islet size distribution of mouse islets following 96 h culture under baseline conditions, regular culture media, or starvation media, normalized to baseline islet diameter. Starvation induces significant islet shrinkage compared with regular culture conditions. **b** Gene expression profiling of pathways associated with VEGF-A regulation and cellular stress. Relative mRNA expression of Vegf-a, p62, Lc3, Becn1, Hif1a, Sp1, and Nrf2 in cultured mouse islets is shown for four conditions: baseline (24 h post-isolation), 96 h in regular media without chimera, 96 h in starvation media without chimera, and 96 h in regular media with chimera treatment. Expression levels are normalized to baseline. **c** Representative intravital confocal images of ACE-transplanted LC3-RFP-EGFP mouse islets at 1 and 4 weeks post-transplantation. Chimera-treated and mock-treated grafts are shown. Functional perfusion is visualized by dextran influx (red) and reflection imaging (gray); the LC3-RFP signal is displayed in scaled intensity. EGFP remained undetectable throughout the experiment. **d** Longitudinal quantification of LC3-RFP signal resolution per islet over time in chimera-treated and mock-treated grafts, expressed as the mean fluorescence intensity per voxel. **e** Quantification of intra-islet vascular volume over time in the same grafts shown in (d), demonstrating accelerated vascular integration in chimera-treated islets. **f** Regression analysis across all graft measurements linking intra-islet vascular content to LC3-RFP burden, revealing an inverse correlation between vascularization and stress marker accumulation (slope = −0.43, *r* = 0.94, *n* = 51 derived from longitudinally tracked islets, *P* < 0.0001). Data are presented as the mean ± SD. Statistical comparisons were performed using one-way or two-way ANOVA and linear regression as appropriate. *P < 0.05, **P < 0.01, ***P < 0.001. **d**, **e**, **f** Each data point represents an individual islet unless otherwise stated

Mouse islets exposed to 96 h of nutrient starvation exhibited significant shrinkage relative to baseline or standard culture conditions, consistent with autophagy activation (Fig. 4a). Starvation also upregulated canonical autophagy and stress genes,^24,28,54–58^ including p62, Lc3, Becn1, Hif1a, Sp1, and Nrf2, alongside VEGF-A (Fig. 4b). In contrast, chimera treatment in nutrient-rich media upregulated VEGF-A to similar or higher levels without activating stress- or autophagy-related genes, indicating that VEGF-A induction can occur independently of nutrient deprivation pathways. Complementary LC3-EGFP-RFP^59^ and LysoTracker imaging confirmed that chimera-treated islets in culture maintained a lower autophagic burden compared with starved controls (Supplementary Fig. 3 To confirm these findings at the protein level, coimmunostaining of HIF-1α and VEGF-A in starved and normally cultured islets revealed marked HIF-1α upregulation in starved islets compared with regular cultured islets. Within starved islets, VEGF-A expression was highest in cells showing the greatest HIF-1α immunoreactivity, confirming stress-coupled transcriptional regulation of VEGF-A at single-cell resolution (Supplementary Fig. 3C). Protein-level assessment of SP1 and NRF2 was not performed in the current study.

In vivo, longitudinal confocal imaging of LC3-RFP-EGFP reporter ACE grafts revealed LC3-RFP puncta in transplanted islets despite glucose-replete aqueous humor conditions (Supplementary Fig. 3D). Chimera-treated grafts exhibited significantly fewer puncta at week 1 compared with mock-treated controls, reflecting a reduced autophagic response during the early avascular period (Fig. 4c). Quantification over time demonstrated faster RFP signal resolution in chimera-treated grafts, with most islets approaching baseline by week 2, whereas untreated grafts retained elevated autophagic signals until week 4 (Fig. 4d). Chimera-treated grafts also exhibited accelerated intra-islet vascularization (Fig. 4e), and regression analysis confirmed an inverse relationship between the LC3-RFP signal and vascular content (slope = −0.43, *r* = 0.94, *n* = 51, longitudinally tracked islets, *P* < 0.0001) (Fig. 4f).

These data indicate that VEGF-A upregulation via the aptamer–saRNA chimera reduces the duration and magnitude of post-transplant autophagic stress, preserves islet architecture, and accelerates functional revascularization. These findings provide a mechanistic link between early VEGF-A induction and improved graft homeostasis during early engraftment in islets. Together, these findings suggest that the primary benefit of chimera-mediated VEGF-A induction lies in shortening the avascular phase following transplantation rather than increasing the ultimate vascular capacity of the graft. To determine whether this accelerated stress resolution reflects earlier functional reperfusion and altered revascularization kinetics in a translational setting, we next examined longitudinal vascular integration of human islets in vivo.

### Accelerated revascularization kinetics of human islet grafts following chimera treatment

**Fig. 5:**
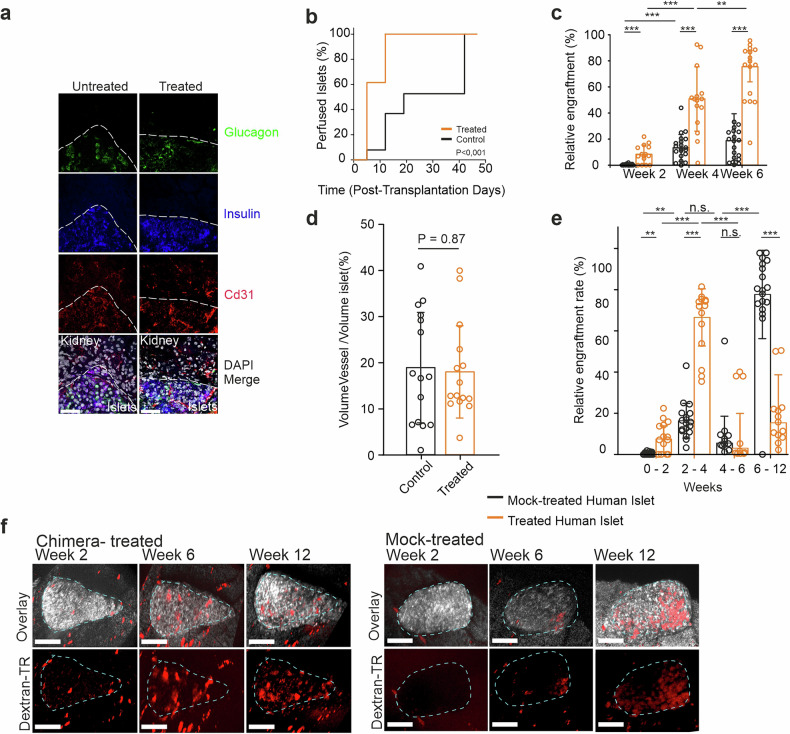
Aptamer–saRNA chimera accelerates islet revascularization without affecting long-term vascular endpoints. **a** Confocal immunofluorescence images of chimera-treated and mock-treated human islets transplanted under the kidney capsule one week post-transplantation. Glucagon (green), Insulin (blue), Cd31 (red), and DAPI (gray). Scale bar = 50 μm. **b** Kaplan–Meier analysis showing time to first detectable functional reperfusion for individual ACE-transplanted islets, defined by intravascular dextran influx. Data are normalized to the total number of transplanted islets per group. Chimera-treated islets exhibited significantly earlier reperfusion than mock-treated controls (*P* < 0.001, log-rank test). **c** Relative vascular engraftment over the first 6 weeks post-transplantation. Vascular content is normalized to the individual islet’s vascular endpoint. Lines represent mean engraftment trajectories, and individual data points correspond to longitudinally tracked single islets. **d** Endpoint intra-islet vascular content at 12 weeks post-transplantation. No significant difference is observed between chimera-treated and mock-treated islets (*P* = 0.87, Student’s *t* test), indicating comparable long-term vascularization. **e** Relative change in intra-islet vascularization between consecutive imaging intervals, highlighting distinct revascularization dynamics. Chimera-treated islets undergo most vascular integration between weeks 2–4, whereas mock-treated islets show delayed revascularization, mainly between weeks 6 and 12. **f** Representative top-view confocal projections of transplanted islets at weeks 2, 6, and 12 post-transplantation, illustrating earlier vascular network formation in chimera-treated grafts, supporting enhanced graft integration. Data are presented as the mean ± SD ; *n* = 15 islets/group from 3 mice (**b**–**f**). **c**, **e** Statistical comparisons were performed using two-way ANOVA; significance is indicated as **: *P* < 0.01 and ***: *P* < 0.001, n.s. No significance

To assess whether the accelerated stress resolution observed in mouse islets translates into earlier functional reperfusion in a clinically relevant setting, we transplanted chimera-treated or mock-treated human islets into the ACE of immunodeficient Rag1⁻^/^⁻ mice and monitored vascular integration longitudinally using intravital imaging (Fig. 5).

To first establish whether the proangiogenic effect of chimera treatment extends to human islets at an established transplantation site, chimera-treated and mock-treated human islets were transplanted under the kidney capsule. After one week, we assessed the graft sections by confocal immunostaining. Chimera-treated grafts exhibited markedly enhanced CD31⁺ vascularization, while mock-treated grafts showed sparse vascular ingrowth, limited to the outer region of the graft (Fig. 5a). These data confirm that the proangiogenic effect of chimera priming is operative in human islets at a transplantation site routinely used in preclinical islet research.

Initial graft reperfusion was quantified as the first detectable intravascular dextran influx within individual islets. Kaplan–Meier analysis demonstrated a marked acceleration of functional reperfusion in chimera-treated grafts, with all treated islets exhibiting perfusion by day 12 post-transplantation, whereas mock-treated grafts required up to 42 days to reach comparable levels (Fig. 5b).

To capture revascularization kinetics independent of endpoint variability, intra-islet vascular content was normalized to each graft’s individual vascular endpoint and tracked longitudinally over the first six weeks (Fig. 5c). Chimera-treated islets displayed a rapid and coordinated increase in vascular integration, whereas mock-treated grafts showed delayed and heterogeneous engraftment trajectories.

Despite these pronounced kinetic differences, quantification of intra-islet vascular content at 12 weeks post-transplantation revealed no significant difference between chimera-treated and mock-treated grafts (Fig. 5d), indicating that VEGF-A induction advances the timing, but not the extent, of long-term vascularization.

Analysis of relative changes in vascular content between consecutive imaging intervals further highlighted distinct phases of vascular integration (Fig. 5e). Chimera-treated islets underwent the majority of vascular ingrowth between weeks 2 and 4, whereas revascularization in mock-treated grafts occurred later and more gradually, predominantly between weeks 6 and 12.

Representative top-view confocal projections illustrate these dynamics, with chimera-treated grafts exhibiting early and organized vascular network formation, while mock-treated islets remain sparsely perfused during early time points (Fig. 5f).

Collectively, these data demonstrate that aptamer–saRNA-mediated VEGF-A induction accelerates functional reperfusion and compresses revascularization kinetics of human islet grafts without altering long-term vascular endpoints, consistent with a mechanism centered on shortening the avascular post-transplantation phase. To assess the functional secretory correlate of this accelerated engraftment, circulating C-peptide was measured longitudinally in kidney capsule transplant recipients. Chimera-treated recipients showed higher plasma C-peptide levels than mock-treated controls after one week, consistent with earlier restoration of β-cell secretory function following engraftment (Supplementary Fig. 4).

Accelerated reperfusion and compressed revascularization kinetics are expected to be particularly relevant under conditions of increased metabolic demand. While long-term vascular endpoints were comparable between groups, earlier restoration of blood supply may enhance graft resilience when islets are challenged by hyperglycemia. To determine whether accelerated revascularization translates into improved functional outcomes, we next evaluated glucose control and graft performance in a diabetes-induction model.

### Chimera-primed human islets ameliorate STZ-induced diabetes in Rag1^‒/‒^ mice

**Fig. 6:**
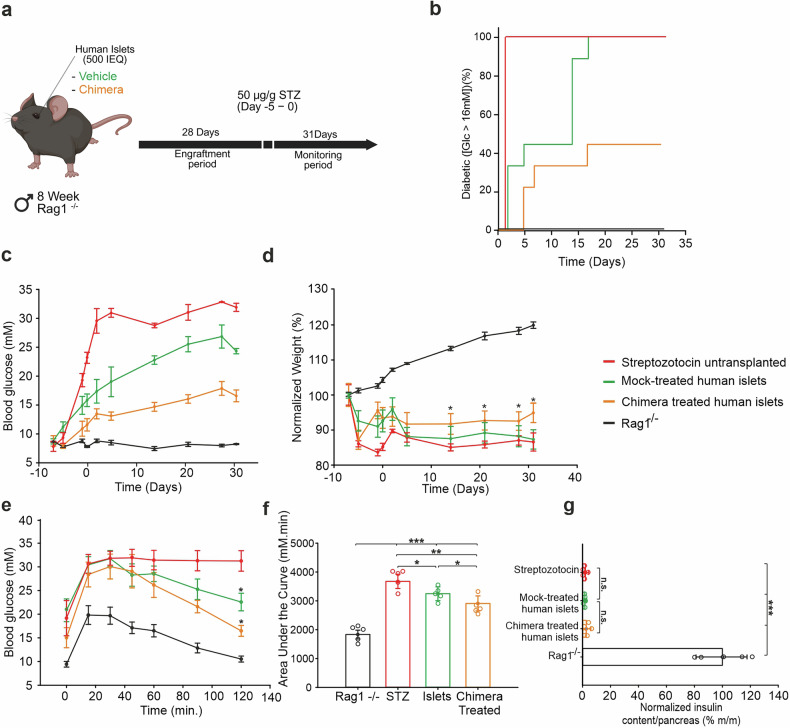
Aptamer–saRNA chimera-mediated VEGF-A induction improves human islet transplantation outcomes. **a** Experimental scheme: 8-week-old male Rag1^‒/‒^ mice were transplanted with 500 IEQ of human islets into the ACE and allowed to engraft for 4 weeks. Diabetes was induced using a 5-day low-dose STZ regimen, followed by longitudinal blood glucose monitoring and an IPGTT at the end of the 31-day tracking period. Created with BioRender.com. **b** Kaplan–Meier analysis of hyperglycemia onset, defined as blood glucose >16 mM. Chimera-treated islet recipients exhibit delayed diabetes onset compared with mock-treated controls (*P* < 0.001, log-rank test). **c** Longitudinal blood glucose measurements over the study period, showing improved glycemic control in chimera-treated grafts relative to controls. **d** Body weight trajectories of transplanted mice, demonstrating preserved weight maintenance in (**b**). **e** Intraperitoneal glucose tolerance test (IPGTT) at day 31 post-transplantation, showing enhanced glucose clearance in chimera-treated mice. **f** Area under the curve (AUC) analysis of IPGTT in (**e**), confirming significantly improved glucose tolerance in chimera-treated recipients. **g** Pancreatic insulin content at endpoint, normalized to total protein, indicating superior β-cell function in chimera-treated grafts. Data are presented as the mean ± SEM (**c**–**f**); mean ± SD in (**g**); for **c**, **d**: *n* = 5 (Rag1^−^^/^^−^ healthy/STZ controls), *n* = 9 mice across three independent donor preparations (Supplementary Table 1) (chimera-treated and mock-treated islet groups); for **e**, **f**, **g** *n* = 5 for all groups. Statistical comparisons (**c**–**g**): **b** log-rank test; **c**, **d**, **e**, **f**, **g** two-way or one-way ANOVA with appropriate post hoc tests. Significance is denoted as *: *P* < 0.05, **: *P* < 0.01, ***: *P* < 0.001, n.s. no significant difference

To determine whether accelerated revascularization translates into functional benefit under metabolic stress, we evaluated the performance of chimera-treated human islets in a diabetes induction model (Fig. 6). Human islets (500 IEQ) were transplanted into the ACE of immunodeficient Rag1^‒/‒^ mice and allowed to engraft for four weeks prior to induction of diabetes using a five-day low-dose STZ regimen (Fig. 6a).^60^ Blood glucose levels were monitored longitudinally for 31 days, and metabolic function was assessed by intraperitoneal glucose tolerance testing at the study endpoint.

Kaplan–Meier analysis revealed a marked delay in the onset of hyperglycemia in recipients of chimera-treated islets compared with mock-treated controls (Fig. 6b). While STZ-only mice rapidly developed diabetes within two days of induction and all mock-treated graft recipients eventually became hyperglycemic, less than half of the mice receiving chimera-treated islets reached the hyperglycemic threshold (>16 mM) during the monitoring period (*P* < 0.001).

Consistent with this delay, longitudinal blood glucose measurements demonstrated improved glycemic control in chimera-treated graft recipients relative to mock-treated controls (Fig. 6c). Whereas mice receiving untreated islets exhibited a progressive rise in blood glucose levels following STZ treatment, chimera-treated recipients maintained lower glucose concentrations throughout the observation period. Although normoglycemia was not fully restored, glucose levels remained substantially reduced compared with untreated graft recipients, consistent with the use of a submarginal graft mass.

Body weight trajectories further supported improved metabolic resilience in chimera-treated mice (Fig. 6d). All STZ-treated groups experienced weight loss following diabetes induction. However, chimera-treated graft recipients showed significantly attenuated weight loss compared with the STZ-only and mock-treated groups, despite incomplete recovery to baseline levels.

At the endpoint, intraperitoneal glucose tolerance testing revealed enhanced glucose clearance in mice receiving chimera-treated human islets compared with mock-treated controls (Fig. 6e). Quantitative analysis of glucose excursions by area under the curve confirmed significantly improved glucose tolerance in the chimera-treated group, while remaining distinct from healthy controls (Fig. 6f).

Finally, analysis of pancreatic insulin content demonstrated that all STZ-treated groups exhibited similarly reduced endogenous insulin levels relative to healthy mice (Fig. 6g), confirming effective ablation of host β-cells. This indicates that the observed metabolic improvements in chimera-treated mice derive from the functional contribution of the transplanted human islets rather than residual endogenous insulin production.

Together, these findings show that VEGF-A induction via aptamer–saRNA chimeras enhances the functional resilience of human islet grafts under diabetic stress, delaying hyperglycemia onset and improving glucose handling without fully correcting systemic metabolism. These results are consistent with accelerated graft reperfusion and support the conclusion that early vascular integration confers a meaningful functional advantage in marginal human islet transplantation.

## Discussion

Islet transplantation is constrained not only by donor availability but also by a convergence of biological stresses during early engraftment, including inflammatory injury, mechanical damage and a prolonged avascular period that imposes diffusion-limited oxygen and nutrient supply.^1,3,20^ Although these insults arise through distinct mechanisms, they converge on a shared requirement for timely vascular integration. During this period, vascular ingrowth is initiated largely through stress-induced VEGF-A expression, triggered by hypoxia, nutrient deprivation, and oxidative signaling via pathways including HIF1α,^57^ SP1,^56^ and NRF2.^58^ While adaptive, this mechanism delays revascularization and prolongs a metabolically compromised state in which autophagy becomes essential for islet survival,^24,27^ potentially rendering grafts more vulnerable to secondary metabolic stress during recovery, such as hyperglycemia. Consistent with this framework, untreated grafts exhibited sustained LC3 accumulation until functional perfusion was established, indicating prolonged stress exposure during early engraftment.

In this study, we demonstrate that transient VEGF-A induction via a β-cell-targeted aptamer–saRNA chimera shifts this paradigm by advancing the timing of vascular integration rather than increasing its final extent. Across mouse and human transplantation models, chimera-treated islets consistently achieved earlier functional reperfusion, as quantified by dextran influx and longitudinal intravital imaging (Figs. 2, 5). Importantly, while revascularization occurred more rapidly, endpoint vascular density remained comparable between treated and untreated grafts, indicating that early VEGF-A priming accelerates physiological vessel recruitment without promoting excessive or aberrant angiogenesis, consistent with the heterogeneous and delayed revascularization observed in mock-treated grafts across both models (Figs. 2f, 5e). Mechanistically, our data support a model in which accelerated reperfusion shortens the duration of transplantation-associated stress rather than directly suppressing stress pathways. In vitro starvation experiments showed that nutrient deprivation activates autophagy and stress-responsive transcriptional programs, including pathways known to regulate VEGF-A expression. In contrast, chimera treatment upregulated VEGF-A under standard culture conditions without inducing autophagic or stress-gene signatures (Fig. 4). In vivo, longitudinal imaging of LC3-RFP-EGFP reporter islets revealed that chimera-treated grafts resolved autophagic burden more rapidly, and regression analysis demonstrated a strong inverse correlation between intra-islet vascular content and LC3 accumulation. These findings indicate that restoration of nutrient and oxygen supply, enabled by earlier vascularization, drives stress resolution, rather than a direct effect of the chimera on autophagic machinery. Importantly, equivalent endpoint vascular density between groups does not imply equivalent graft mass at the time of post-engraftment metabolic challenge. Mock-treated grafts underwent a more protracted avascular phase, accumulating greater autophagic burden (Fig. 4c–f), and lost more functional β-cell mass before vascular integration was achieved, even as the eventual vascular bed approached a comparable endpoint (Fig. 5d). The differential resilience to metabolic challenge observed in chimera-primed grafts therefore reflects preservation of greater functional β-cell mass through earlier restoration of nutrient and oxygen exchange. The four-week pre-engraftment window in the human cohort (Fig. 6), well past the chimera-active window established in Fig. 1e, f, indicates that the functional benefit at the time of metabolic challenge derives from preservation through the avascular phase rather than from ongoing chimera activity.

The functional benefits observed in marginal-mass mouse and human models support the translational relevance of the approach. The use of STZ-induced diabetes and immunodeficient recipients provides a controlled setting to isolate the contribution of early revascularization to graft function, but does not replicate the full complexity of the clinical environment. STZ ablates mouse β-cells rapidly and reproducibly but lacks the autoimmune component of clinical T1D, and immunodeficient recipients remove allorejection, meaning the functional advantages demonstrated here were obtained under conditions more permissive than clinical practice. These are deliberate model choices designed to amplify the signal of interest rather than introduce confounds. Validating the approach in immunocompetent and autoimmune settings remains an important next step. The marginal-mass design was chosen specifically to amplify differences between groups; graft sizes (50 mouse islets in Fig. 3; 500 IEQ human islets in Fig. 6) were deliberately set below the threshold for full metabolic rescue. Consistent with this design, recipients of chimera-primed grafts achieved improved glycemic control without fully restoring baseline body weight, indicating that the engrafted β-cell mass approached but did not reach complete sufficiency. This regime, in which differential graft preservation is most noticeable, provides the most demanding test of the priming effect. The portal vein remains the leading clinical site for islet transplantation, but its association with the instant blood-mediated inflammatory reaction (IBMIR)^9^ has motivated continued investigation of extrahepatic sites, including the kidney capsule, omental, subdermal and intramuscular implantation,^11,12^ and the recently described anterior rectus sheath,^4^ each presenting a distinct vascular and inflammatory microenvironment. A common feature across these extrahepatic sites is the prolonged avascular phase that follows transplantation, the duration and severity of which substantially shape graft outcomes. Because the priming approach described here targets this step directly, pre-engraftment priming may complement ongoing efforts to optimize transplantation site selection. Our kidney capsule data (Fig. 5a, Supplementary Fig. 4) indicate that the priming effect is operative beyond the ACE in another extrahepatic setting, supporting the view that the underlying mechanism of accelerated vascularization is not specific to a single anatomical site. The portal vein, by contrast, presents site-specific challenges beyond the avascular phase, and translation to that setting will require approaches that extend beyond VEGF-A induction. Glycemic control trajectories and pancreatic islet insulin content, which confirmed effective β-cell ablation across all STZ-treated groups, were used as the primary functional readouts and are established surrogates in marginal-mass transplantation models.^13^ Nevertheless, future studies incorporating serial C-peptide measurements would strengthen the attribution of metabolic recovery to graft function and enable more precise dose-response modeling. Notably, the absence of increased endogenous insulin content across STZ-treated groups confirms that the observed metabolic benefits are graft-derived, which remains an important internal control supporting the validity of the functional conclusions drawn. While the in vivo LC3-RFP reporter data provide direct protein-level evidence of reduced autophagic burden in chimera-treated grafts, western blot-level quantification of autophagy and stress marker proteins in cultured islets was not the primary focus of this study, which is centered on functional vascular and metabolic outcomes rather than mechanistic dissection of autophagic pathways.

The aptamer–saRNA architecture offers several properties that support translation beyond the current experimental models. Unlike viral^29^ or non-viral^31^ vectors, tethered growth factors,^34^ or sustained mRNA delivery,^37^ the chimera provides transient transcriptional activation within the physiological range of endogenous VEGF-A, avoiding prolonged overexpression that can disrupt vascular architecture. Cell targeting is specified by the aptamer rather than viral tropism, making the architecture inherently modular, i.e., the targeting moiety can be exchanged to direct saRNA delivery to alternative cell types, including engineered or stem-cell-derived β-cell replacements.^5,6,61^ Stem cell-derived islet replacement has now achieved clinical proof of concept,^6^ and recent work has further demonstrated the feasibility of autologous patient-derived stem cell-derived islets.^4^ Because these approaches currently rely on costly small-scale manufacturing per patient, technologies that preserve a greater fraction of the engrafted graft mass are particularly relevant to reducing the per-patient cell requirement. The engraftment stresses targeted by the priming strategy described here, therefore, apply equally to donor-derived and stem cell-derived islet grafts,^62,63^ positioning the chimera as a candidate complement to these emerging cell sources. The non-integrative mechanism of saRNA^46,64^ delivery further enhances translational compatibility by avoiding permanent genetic modification. Whether these advantages translate to immunocompetent allograft settings remains an open question. Combining angiogenic priming with immune-protective or encapsulation strategies^10,65^ is a logical next step that could address graft durability beyond the avascular window. Longer-term vascular remodeling studies and validation across clinical transplantation sites will be an important focus in future evaluation. We further note that the ACE is not a well-accepted clinical transplantation site yet.^11,14^ Nevertheless, we are in clinical trials in Europe,^15^ USA^16^ and China evaluating its potential, with the first T1D patients having recently been transplanted (unpublished data). The ACE has also been used to demonstrate in vivo maturation of stem cell-derived islets,^63^ supporting its potential applicability across both donor-derived and stem cell-derived transplantation strategies. Together, these directions define a tractable path from proof-of-concept to a manufacturable, ex vivo priming protocol compatible with existing islet processing workflows.

In summary, these findings establish that transient, targeted transcriptional activation of VEGF-A is sufficient to accelerate early vascular integration, shorten transplantation-associated stress, and preserve functional β-cell mass in marginal graft settings. By increasing the rate rather than the extent of revascularization, VEGF-A priming improves the timing of graft integration without perturbing long-term vascular endpoints. Beyond defining a tractable biological principle, this work introduces an RNA-guided priming strategy that is compatible with donor islets, engineered cellular therapies and stem cell-derived replacement tissue. As regenerative approaches in diabetes move toward curative intent with increasingly limited graft material, technologies that improve the efficiency and reliability of early engraftment will be essential. Rather than replacing intrinsic adaptive responses, RNA-guided priming preconditions transplanted cells to better withstand the physiological stresses associated with engraftment and integration.

## Materials and methods

### Mice

All experiments were performed following the Karolinska Institute’s guidelines for care and use of animals in research and reported following the Animal Research Reporting of In vivo Experiments (ARRIVE) guidelines for animal studies. All procedures were approved by the Animal Review Board at the Court of Appeal of Northern Stockholm, under ethical permit number 8822-2020. All mice were housed in a temperature- and humidity-controlled room with 12-h light/12-h dark cycles with food and water ad libitum. Six-to-eight-week-old C57BL/6J mice as well as RAG1−/− mice and C57BL/6-Tg(CAG-RFP/EGFP/Map1lc3b)1Hill/J were purchased from Jackson Laboratories (Germany and United States, respectively). For mouse studies, islets were isolated from C57BL/6J male mice at 3–5 months of age. All mice were fed with normal chow. Hyperglycemia was induced with streptozotocin, (STZ; Sigma). We used 150 g/kg in “single high dose experiments” and gave immune-compromised mice 50 g/kg of STZ for 5 consecutive days in “low dose experiments” as they do not tolerate higher dose levels of STZ well. Blood glucose and body weight were monitored daily after the first STZ intraperitoneal injection until hyperglycemia was established (>16.0 mM glucose). Mice were not fasted when given STZ intraperitoneally. Once injected, the body weight and blood glucose were measured every 5–7 days to minimize handling stress. Glucose tolerance tests of mice used in transplantation experiments were assessed at the end of the monitoring period before euthanizing them. Blood glucose concentrations in mice were obtained using the Contour plus monitoring system (Contour), which allows readings up to a maximum of 35.0 mM glucose. Values exceeding this threshold were recorded as 35.0 mM. Insulin was measured by AlphaLISA immunoassay (PerkinElmer) according to the manufacturer’s instructions. Pancreatic protein content was measured using Qubit (ThermoFisher) according to the manufacturer’s instructions.

### Islet transplantation

Islet transplantation was performed under general anesthesia using 2% isoflurane (Baxter, USA) in medical-grade oxygen. Mice were positioned in a custom-built head holder (Narishige, Japan) to ensure stability and optimal isoflurane delivery during the procedure. For transplantations into the ACE, a self-sealing corneal incision (1–1.5 mm) was made using a MICRO FEATHER® ophthalmic microscalpel (Phaco, Germany). For imaging-based studies of revascularization dynamics using reporter islets, 2–12 islets were loaded into a custom-fabricated glass micropipette and gently positioned on the iris surrounding the pupil. Micropipettes were manufactured in-house from borosilicate glass capillaries (WPI, USA) using a micropipette puller and beveller (Sutter Instruments, USA), and sharp edges were softened with a lighter to minimize tissue damage during injection. For metabolic transplantation experiments, islets were seeded on the iris to the opposite side of the incision relative to the pupil and gently guided into the iridocorneal angle to avoid interference with the pupil. In syngeneic transplantations, 50 islets were manually picked and transplanted. In human islet xenotransplantations, approximately 500 islet equivalents (IEQ), as determined using an islet counter (Biorep, USA), were transplanted per mouse. At the conclusion of the procedure, intraocular pressure was carefully released to minimize the risk of graft loss via backflush. For transplantations in the kidney, we shaved the left flank of each recipient mouse and sterilized the skin with iodine solution. We made a small incision above the kidney in the skin and subsequently in the omentum. The kidney was gently squeezed outside while keeping hydrated with 0.9% saline (Baxter, USA). A small incision was made with a 30 G needle in the kidney capsule, and 100–150 islets were seeded in the subcapsular space. The needle cut was cauterized, and the kidney was gently repositioned in place. The omental fleece was sutured, and the skin was closed with an agraffer (TG-Instruments AB, Sweden). For each transplantation method, post-operative analgesia was provided via subcutaneous injection of 2 µg of Temgesic (RB Pharmaceuticals Limited, UK). For kidney capsule transplantations, we provided this dose every 12 h for 72 after the operation.

### In vitro islet imaging

In vitro confocal imaging was performed on either whole live islets, on fixed islets or islet explants. Islets were fixed for 30 min of 10% TRIS-buffered formalin treatment. To permeabilize the fixed islets, a 1% Triton X-100 (Sigma) PBS (GIBCO) solution was used. Islets were washed and incubated in 2% BSA (Sigma) in PBS solution. The islets were stained in 300 µl of primary antibodies (guinea pig anti-insulin polyclonal, DAKO; rabbit anti-glucagon, polyclonal, Biogenix; rat anti-mouse VEGF-A, LSBio; and mouse anti-human VEGF-A, R&D Systems). Live islets were labeled with Lysotracker Deep Red (ThermoFisher, USA) by incubation in media for 1 h and then moved to fresh media for at least 2 h to mitigate diffusion-based artifacts. A TCS SP8 (Leica, Germany) confocal microscope with a 63× and 85% glycerol in water immersion objective was used in these studies.

### Intravital imaging

Islet grafts were imaged between 1 and 12 weeks post-transplantation by intravital confocal microscopy. To visualize islet vascularization, animals were anesthetized with 2% isoflurane (Baxter, USA). One hundred microliter of PBS solution containing 1 mg/ml (mouse islets) and 5 mg/ml (human islets) of 70 kDa Texas Red- or 10 kDa Alexa Fluor 647-conjugated dextran (ThermoFisher, Fisher Scientific, USA) was injected intravenously prior to imaging. Z-stacks of 3–5 μm were acquired for every islet graft (reflection: 561 ± 3 nm, fluorescence: Ex: 594 nm or 633 nm, Em: 600–700 nm or 655–750 nm, respectively). Images of islet grafts were obtained by a Leica SP5 system with a 25× objective (N.A.0.95, Leica Microsystems, Germany). As an immersion medium between the objective and the eye, we used Viscotears (Laboratoires Théa, France).

### Image analysis

Unprocessed original z-stacks were used in all quantifications of in vivo re-vascularization. Islet volumes were measured using backscatter reflection signals with Volocity 6.0 image analysis software (PerkinElmer, USA). Dextran-labeled islet vessels were identified using manual thresholding to correct for cornea haziness caused by the relative position of the islets on the iris (the bend of the eye makes the cornea thicker at the edge of the iris), and structures smaller than 6000 voxels were excluded. Their volumes (“Find Object” function) were measured using Volocity. Vascularization was quantified as the proportion of vascular volume within each islet relative to its total volume, and then normalized based on the vascularization level observed at the 12-week endpoint. The islet volume was determined by the “FindObject” function in the reflective signal channel, using the ROI function.

### Islet isolation and culture

Mouse islets were isolated by microinjecting the pancreas with a 27 G needle with collagenase P, 1.0 mg/ml in HBSS. Injected pancreases were then digested under light shaking conditions for 20 min in a glass vial. After digestion, the pancreas was visually checked for total digestion, after which the digest was quenched with an ice-cold 2% BSA (Sigma) HBSS solution. The digest was passed five times through a long 14 G needle, after which the digest was spun down at 300 × *g* for 5 min. The supernatant was discarded, and the pellet was homogenized in 1077 Histopaque solution (Sigma). Then, 1077 histopaque solution was added to this homogenate to form the bottom layer, after which 10 ml of 2% BSA HBSS solution was added without disrupting the formed layers. This was centrifuged at 1700 × *g* for 20 min (acceleration and brake off). After centrifugation, the top fraction was collected and centrifuged for 5 min at 300 *g* and washed once in 2% BSA HBSS. Islets were reconstituted and then handpicked and cultured for 24 h before starting experiments. Mouse islets were cultured in RPMI 1640 medium with 10% FBS and 1% penicillin–streptomycin (GIBCO), with no more than 70 islets per 35 mm nonadherent culture dish in 10 ml to ensure optimal culture conditions. Human islets were obtained from the University of Uppsala islet isolation core and were cultured in CMRL 1066 media (GIBCO) with 10% FBS and 1% penicillin–streptomycin.

### In vitro VEGF-A ELISA time course

Isolated islets were pooled from different mice for each experiment. Thirty islets from each group and time point were picked into four-well plates containing 1000 µl of full culture medium per well. Chimera was added, which was regarded as the starting point of every time-course experiment. After 48 h, supernatants were collected, spun down and stored at −80 °C. Islets were collected, lysed, and stored at −80 °C in 100 µl M-PER buffer (ThermoFisher, USA) for quantification of DNA contents by the Qubit dsDNA Assay Kit (ThermoFisher, USA) according to the manufacturer’s instructions. Secreted VEGF-A in the supernatants was measured with the respective mouse and human VEGF Quantikine ELISA Kit (R&D Systems, USA), per manufacturer’s instructions.

### qRT-PCR and relative isoform analysis

Islets were prepared as described in the ELISA time course section. When harvesting the islets after the appropriate incubation period, the islets were handpicked and placed in TRIzol and stored at −20 °C until analysis. for analysis. Gene expression was calculated using ΔΔCt with 18S expression (Applied Biosystem) as a reference. For relative isoform analysis experiments, we used SSO advance SYBR Green supermix (Bio-Rad) with the appropriate primers that were specific for the isoforms of mouse and human VEGF-A (Table 1).

**Table 1:** List of oligonucleotides

Name	Sequence
1-717 template	GGAGGACGATGCGGTAATTCTCAGGAGGTGCGGAACGGGATATGGA TTGTTCGCCAGACGACTCGCTGAGGATCCGAGA
M12-3773 template	GGAGGACGATGCGGCAACAAACTAATCAGACACGAGACAGAGAGAT AGATCTGCCAGACGACTCGCTGAGGATCCGAGA
5’-primer	GGGGGAATTCTAATACGACTCACTATAGGGAGGACGATGCG
3’-primer	TCTCGGATCCTCAGCGAGTCGTC
1-717 Scr Template	GGAGGACGATGCGGGGCTCGCTGGAGTACTAATCGTTGACGGAATGG CATTGATAGGACGACTCGCTGAGGATCCGAGA
M12-3773 Scr Template	GGAGGACGATGCGGACAACATAATAGAGGCGCAATAGCAAGCGAGC TACAACACATGACGACTCGCTGAGGATCCGAGA
3’- mVEGFa	TCACATTGAAACACGCGTCGGGTCTCGGATCCTCAGCGAGTCGTC
3’- Scr	CGTAGTGAGCGATTGTCATGGGTCTCGGATCCTCAGCGAGTCGTC
3’-hVEGFA	TATTTGGGACTGGAGTTGCGGGTCTCGGATCCTCAGCGAGTCGTC
mVegf-a Guide (RNA)	UCACAUUGAAACACGCGUCUU
hVEGF-A Guide (RNA)	UAUUUGGGACUGGAGUUGCUU
SCR- Guide (RNA)	CGUAGUGAGCGAUUGUCAUUU
mVEGFA-exon2 Forw	CACCCACGACAGAAGGAGAGCAG
mVEGFA-ex5-8jnc Rev	GCCTTGGCTTGTCACATTTTTCTGGC
mVEGFA-exon6a-7jnc Forw	CCTGGAGCGTTCACTGTGAGCC
mVEGFA-exon7-8jnc Rev	CCTTGGCTTGTCACATCTGCAAGTACG
mVEGFA-exon5-7jnc Forw	GCCAGAAAATCACTGTGAGCCTTG
human VEGF forward	GAGCTTCCTACAGCACAACAAA
human VEGF165 r	GCTTTCTCCGCTCTGAGCA
human VEGF189 r	CCACAGGGAACGCTCCAGGAC
human VEGF121 r	CTCGGCTTGTCACATTTTTC

### Construction and use of the aptamer-saRNA chimera

Aptamer-passenger strand conjugates were produced from dsDNA templates, construed by PCR (TaKaRa) and subsequent T7-RNA polymerase (Durascribe, Lucigen) using a T7 promotor region in the forward 5′ primer, and the 3′ primer was downstream elongated with a three-nucleotide spacer sequence and the appropriate passenger strand (Table 1). Guide strands were then annealed at an equimolar (1:1) ratio in a heat block using the following conditions: 70 °C for 10 min, after which the chimeras were allowed to cool to room temperature for at least 10 min before use. Annealing was confirmed by gel shift electrophoresis on a 2% agarose gel. Whole islets were transfected by adding the relevant or scrambled saRNA-aptamer chimera (5 µg/100 islets for mouse islets and 15 µg/500 IEQ for human islets) full islet media. No washing steps, transfection reagents, or media replacement were applied, except in the chimera uptake assay in whole islets, and the time-course assays in Fig. 1. Islets were kept in this medium overnight until use in experiments, for no longer than 24 h.

### Statistical analysis

All results are based on biological replicates and presented as the means ± SEMs unless indicated otherwise. Statistical calculations were performed using Sigmaplot 14 (Systat software). Data normality was assessed using the Shapiro‒Wilk test. Significant differences between the two groups were assessed with Student’s *t* test. Images of islet grafts were obtained by a Leica SP5 system with a 25× objective (N.A.0.95, Leica Microsystems, Germany). As an immersion medium between the objective and eye, we used Viscotears (Laboratoires Théa, France). Two-way analysis of variance (ANOVA) was used to assess significant differences between more than two groups. The multiple comparisons tests used for analysis are specified in the figure legends. Statistical significance was defined by *P* < 0.05. Statistical significances indicated by asterisks not associated with brackets represent analyses performed between treated islet groups and untreated islet groups or between diabetic mice and their control groups, unless specified in the figure legends. In vivo experiments included cohorts of the size indicated in each figure legend (at least 3 mice per group). In vitro analyses and in vivo experiments were performed at least twice to ensure reproducible conclusions. The log-rank test was used for diabetic onset analysis, followed by all pairwise multiple comparison procedures (Holm‒Sidak method). Data from multiple experiments were averaged unless otherwise indicated in the figure legends. No experimental data points were excluded from the analysis. Sample size was chosen by power analysis using the effect size determined by the literature, pilot experiments or prior experience of the authors.

## Supplementary information

Supplementary Materials for β-cell–targeted RNA activation of vascular endothelial growth factor-a improves islet transplantation

## Data Availability

All data supporting the findings of this study are provided within the main manuscript, figures, and supplementary materials.
