# Supplementary material for: β-cell-targeted RNA activation of vascular endothelial growth factor-A improves islet transplantation
Source: Signal Transduct Target Ther. 2026 Aug 4;11:318. doi: 10.1038/s41392-026-02934-8 (PMC13433912; doi:10.1038/s41392-026-02934-8)
Supplement: Supplementary file 1 [file 41392_2026_2934_MOESM1_ESM.docx]

Supplementary Materials for

β-cell–targeted RNA activation of Vascular endothelial growth factor-A improves islet transplantation

Dimitri Van Simaeys,1,* & Per-Olof Berggren,1,2,3,4

1 The Rolf Luft Research Center for Diabetes and Endocrinology, Karolinska Institutet, Stockholm, Sweden

2 Diabetes Research Institute, University of Miami Miller School of Medicine, Miami, FL, USA

3 West China Hospital, Sichuan University, Chengdu, China

4 Tecnológico de Monterrey, School of Medicine and Health Sciences, Monterrey, NL, Mexico

*Correspondence: Dimitri.Van.Simaeys@ki.se

**This PDF file includes:**

Supplementary Methods: Glucose-stimulated insulin secretion assay, Circulating human C-peptide quantification

Supplementary Text: Development and validation of the in vitro starvation model

Supplementary Figures 1,2, 3, and 4

Supplementary Table 1: Overview of human donor material used per figure, with basic biometrics

**Materials and Methods**

**Supplementary Methods**

**Glucose-stimulated insulin secretion assay**

Glucose-stimulated insulin secretion (GSIS) was assessed in mouse islets 96h after chimera treatment. Groups of ten islets per replicate were transferred to 1.5 ml microcentrifuge tubes and pre-incubated for 1h at 37°C in Hanks Buffered Salt Solution buffer ( HBSS, with 1% BSA, pH 7.4) supplemented with 3 mM glucose to establish basal secretory tone. Islets were subsequently incubated sequentially for 1h each under the following conditions: basal stimulation (3 mM glucose), high glucose stimulation (11 mM glucose), return to basal (3 mM glucose washout), and membrane depolarization (3 mM glucose + 25 mM KCl). Supernatants were collected at each step and stored at −80°C pending analysis. Insulin concentrations were quantified using the AlphaLISA Mouse Insulin Detection Kit (PerkinElmer) according to the manufacturer's instructions. Briefly, samples and acceptor-conjugated anti-insulin antibody were combined in a 96-well OptiPlate and incubated for 60 min at room temperature, followed by addition of streptavidin-coated donor beads and a further 30 min incubation under subdued light. Plates were read on an EnVision multilabel plate reader (PerkinElmer) using standard AlphaLISA settings. Insulin concentrations were interpolated from a recombinant mouse insulin standard curve run in parallel on each plate. Results are expressed as insulin secretion normalized to islet number.

**Circulating human C-peptide quantification**

Plasma human C-peptide was measured in immunodeficient recipient mice transplanted with human islets under the kidney capsule. At one week post-transplantation, mice were terminally anaesthetised and blood was collected by cardiac puncture into EDTA-coated tubes. Plasma was isolated by centrifugation at 2000 × g for 10 min at 4°C and stored at −80°C until analysis. Human C-peptide concentrations were quantified using the Human C-Peptide ELISA Kit (Crystal Chem) according to the manufacturer's protocol. Briefly, plasma samples were thawed on ice and pre-incubated with the provided anti-human C-peptide antibody-HRP conjugate solution for 120 min at room temperature with gentle shaking in the antibody-coated 96-well plate Following five washes with wash buffer, TMB substrate solution was added and the colorimetric reaction was allowed to develop for 15 min at room temperature in the dark before termination with stop solution. Absorbance was measured at 450 nm with reference correction at 630 nm on a microplate spectrophotometer. Results are expressed as pg/µl plasma. Statistical comparison between chimera-treated and mock-treated groups was performed using the Student's *t*-test.

**Supplementary Text**

**Supplementary Note: Development and validation of the in vitro starvation model**

To model the nutrient-limited conditions experienced by islets during the avascular post-transplantation period, we established an in vitro starvation protocol using isolated mouse islets and characterized autophagic activity, stress pathway activation, and hypoxia-responsive gene expression across a 96h culture window.

Autophagic flux was assessed using islets derived from LC3-RFP-EGFP tandem reporter mice, which exploit the differential pH stability of the two fluorophores: autophagosomes appear as yellow (RFP⁺EGFP⁺) puncta, while autolysosomes appear as RFP-only puncta due to EGFP quenching in the acidic lysosomal environment. The near-complete absence of detectable EGFP signal across all conditions indicates that LC3-positive structures are predominantly in the autolysosomal stage, consistent with active autophagic flux rather than a block at the autophagosome level. We note that definitive flux quantification requires inhibitor-based approaches, such as bafilomycin A1 treatment, which were beyond the scope of this imaging setup. LysoTracker Deep Red co-staining was used to mark lysosomal compartments. In normally cultured islets, minimal overlap was observed between the LysoTracker signal and the reflection channel — used here as a proxy for insulin granule density — consistent with low basal rates of granule-lysosome fusion. In starved islets, increased co-localisation between the reflection signal and LysoTracker-positive areas was observed alongside regions of RFP-LC3 overlap, indicative of enhanced starvation-induced nascent granule degradation (SINGD) and autophagic activity, consistent with the unconventional autophagic regulation described in pancreatic β-cells.

To confirm activation of hypoxia-responsive pathways at the protein level, we performed co-immunostaining for HIF-1α and VEGF-A in islets cultured under normal and starvation conditions. Starved islets displayed an overall higher HIF-1α signal relative to normally cultured controls. Within starved islets, VEGF-A upregulation was observed at discrete cell-level regions, consistent with selective induction in a subset of β-cells. Notably, the cells showing the highest VEGF-A signal were also those with the greatest HIF-1α immunoreactivity, confirming stress-coupled transcriptional regulation of VEGF-A at single-cell resolution and corroborating the mRNA-level findings reported in Figure 4b.

Together, these data validate the starvation model as a faithful in vitro correlate of the nutrient-deprived avascular phase, and provide the mechanistic context for interpreting the gene expression and autophagic flux data presented in Figure 4.

Supplementary Figure 1.

Supplementary Figure 1. Aptamer–saRNA Vegf-a chimera treatment does not impair β-cell secretory function.

Mouse islets were treated with aptamer–saRNA VEGF-A chimera, aptamer-Scr control, or left media-cultured for 96 hours prior to glucose-stimulated insulin secretion (GSIS) assessment. (a) Media insulin secretion measured across sequential stimulation conditions: basal glucose (3 mM), stimulatory glucose (11 mM), washout to basal (3 mM II), and membrane depolarization (25 mM KCl). All three groups showed comparable insulin secretion patterns with preserved stimulation-secretion coupling across conditions. (b) Vegf-a secretion measured in parallel across the same stimulation sequence. Chimera-treated islets exhibited elevated Vegf-a secretion under stimulatory glucose and KCl conditions relative to aptamer-siGFP and media-cultured controls, confirming active Vegf-a induction at the protein level without disruption of the insulin secretory response. Insulin concentrations were determined by AlphaLISA immunoassay (PerkinElmer). All secretion values are normalized to total DNA content, quantified by Qubit 3.0 fluorometric assay from lysates of 8–10 islets per replicate. Mean ± SEM; n = 4.

Supplementary Figure 2.

**Supplementary Figure 2:** Loss of endogenous vasculature in isolated mouse islets.

Whole islet staining of CD31⁺ endothelial signal in cultured mouse islets demonstrates rapid post-isolation vascular regression. Immunofluorescence analysis of insulin, glucagon, and CD31 at 8 h, 24 h, and 48 h reveals progressive endothelial loss, with near-complete disappearance of CD31 signal by 48 h. Scale bar = 20 μm.

**Supplementary Figure 3**

**Supplementary Figure 3: Characterization of islet autophagic activity and stress responses under starvation conditions.**

**(a)** Relative LC3 signal after 96h in vitro under starvation or regular culture conditions, normalized to 24h post-isolation baseline. **(b)** Confocal microscopy of whole mouse islets expressing the tandem LC3-RFP-EGFP reporter, incubated with LysoTracker Deep Red, under baseline, RPMI, and starvation conditions. Cyan arrows indicate sites of autophagic activity. Representative zoomed panels show individual islet cells at higher magnification. **(c)** Whole mount immunofluorescence co-staining for HIF-1α and VEGF-A in islets under normal culture and starvation conditions after 96 h. **(d)** Glucose concentrations in aqueous humor compared with matched blood glucose in ACE-transplanted LC3 reporter mice (n = 7). **(e)** Light reflectance of chimera- or mock-treated ACE-transplanted islets relative to iris signal (n=5/group). Scale bars: (b) whole islet 20 µm, zoomed panel 6 µm; (c) 20 µm; . Data are presented as mean ± SD. N.D. = not detected; n.s. = not significant; *P < 0.05; **P < 0.01; ***P < 0.001, Two-way ANOVA.

**Supplementary Figure 4**

**ure S3**

**Supplementary Figure 4: Circulating human C-peptide in kidney capsule islet graft recipients.**

Plasma human C-peptide levels measured one week post-transplantation in immunodeficient mice receiving 700–1000 IEQ of chimera-treated or mock-treated human islets under the kidney capsule. Chimera-treated recipients showed markedly higher circulating human C-peptide compared with mock-treated controls, indicating earlier restoration of β-cell secretory function following engraftment. Each data point represents an individual animal. Data are presented as mean ± SD. *P* = 0.03, Student's *t*-test.

**Supplemental Table 1.** **Overview of human donors**

| Human Pancreas | Donor Age | Donor Sex | BMI | Hb1c (mmol/mol) | Used in Figure: |
| --- | --- | --- | --- | --- | --- |
| H2516 | 55 | M | 22,4 | 36,0 | 1 |
| H2543 | 50 | M | 25,9 | 39,0 | 1 |
| H2550 | 72 | M | 24,5 | 39,0 | 1 |
| H2555 | 58 | M | 21,8 | 35,0 | 1 |
| H2583 | 49 | M | 23,6 | 33,0 | 5 |
| H2608 | 49 | M | 27,5 | 33,0 | 1; 5 |
| H2610 | 62 | M | 22,2 | 36,0 | 1 |
| H2613 | 65 | M | 22,3 | 38,0 | 6 |
| H2651 | 63 | F | 22,0 | 37,0 | 6 |
| H2660 | 57 | M | 26,1 | 46,0 | 6 |
| H2783 | 70 | M | 27,8 | 38,0 | 5 |
